# Nanocrystalline Cr-Ni Alloying Layer Induced by High-Current Pulsed Electron Beam

**DOI:** 10.3390/nano9010074

**Published:** 2019-01-07

**Authors:** Lingyan Zhang, Ching-Tun Peng, Jintong Guan, Peng Lv, Qingfeng Guan, Ruifeng Lu

**Affiliations:** 1 Department of Applied Physics, Nanjing University of Science and Technology, Nanjing 210094, China; zhanglingyan1115@hotmail.com (L.Z.); guanjt1224@gmail.com (J.G.); 2 School of Materials Science and Engineering, Jiangsu University, Zhenjiang 212013, China; ctp224@uowmail.edu.au (C.-T.P.); guanqf@ujs.edu.cn (Q.G.)

**Keywords:** high-current pulse electron beam (HCPEB), surface alloying, microstructure, tribological property, corrosion resistance

## Abstract

In this investigation, chromium (Cr) was adopted as an alloying element on a nickel substrate, and the alloying process was materialized via high-current pulsed electron beam (HCPEB) irradiation. X-ray diffraction (XRD), scanning electron microscopy (SEM) and transmission electron microscopy (TEM) were also conducted for microstructure characterization. The results showed that after HCPEB irradiation a nanocrystalline Cr-Ni alloying layer was formed and numerous dislocations were generated, resulting in a great deal of diffusion paths for Cr elements. Moreover, properties including hardness, wear and electrochemical performance were significantly improved after HCPEB irradiation, which was mainly due to the formation of the nanocrystalline Cr–Ni alloying layer. In addition, each strengthening mechanism that contributed to the hardness of the HCPEB-irradiated sample was mathematically analyzed, and solid solution strengthening was found to be of great importance.

## 1. Introduction

Nickel-based alloys not only have a unique corrosion resistance (even at high temperatures) [1,2], but they also hold several advantages, including high strength, good ductility, and the excellent abilities of smelting, casting, cold- or hot-deformation, forming and welding. These merits allow Ni-based alloys to be widely applied in the fields of petrochemistry, energy, marine and aerospace [3]. Nowadays, with the advancement of high temperature alloys, these alloys have a trend whereby the ratio of refractory metal is increased while the Cr ratio is reduced. The purpose of the Cr reduction is to stabilize the alloys, however it degrades the resistances of oxidation and corrosion. On the other hand, for Cr-base alloys elements such as titanium (Ti), iron (Fe), molybdenum (Mo), and tungsten (W) will only exhibit large solubility in Cr at very high temperatures [4], and at low temperatures either form topologically close-packed (TCP) phases (e.g., in Cr–Ti, Cr–Fe) or a very broad miscibility gap (e.g., in Cr–Mo, Cr–W), resulting in a very small solubility in Cr. Consequently, as compared to Cr-based alloys, Ni-based alloys are more widely used.

In most cases, an alloy’s failures generally begin at the surface of the materials. Therefore, surface properties such as hardness, wear and corrosion greatly affect the service life of high temperature alloys [5,6,7]. By modifying the surface, these properties can be drastically improved while maintaining the outstanding original properties of the substrate materials. As a result, a Cr alloying treatment on an Ni substrate was proposed in the current study. 

High-current pulsed electron beam (HCPEB) irradiation, a novel surface-modifying technique, is able to optimize the surface properties by adjusting several parameters such as electron energy, pulse duration and pulse number. During the HCPEB process, as the high energy beam irradiates the material surface, the effects of both rapid heating/cooling and induced thermal-stress field generate various microstructures (e.g., nano-crystallite, amorphous, crystal defects, etc.) [8,9,10,11]. The HCPEB has been utilized in some surface alloying studies, but most of these studies are steel-related [10,12,13]. Consequently, in the current investigation, the feasibility of using HCPEB as a surface-modifying approach was practiced through alloying Cr powder onto Ni substrate.

## 2. Experiments

A pure nickel plate was cut into a 10 × 10 × 10 mm cube and annealed to 700 °C for 4 h. Mechanical grinding and polishing was then performed on one surface of the Ni cube. An organic binder was made up with the ratio of one adhesive (nitrocellulose lacquer) to two diluents (isoamyl acetate). The binder was then mixed with Cr powders (30~40 μm particle size with 99.9% in purity) to form a slurry based with the proportion of 0.1~0.15 g mL^−1^. Afterwards, the slurry was sprayed on the polished Ni surface to form a Cr powder coating 0.05~0.1 mm in thickness, which is described as a Cr–Ni sample hereafter throughout this study. Afterwards, a “HOPE-I” type HCPEB was adopted to irradiate the Cr–Ni samples with 10 and 20 pulses, respectively. The parameters of the HCPEB are given in Table 1. 

Phase identification was carried out by a Rigaku D/max-2500/pc type X-ray diffraction (Rigaku Corporation, Tokyo, Japan) with a CuKα radiation, a NaI crystal scintillation counter detector, a 285 mm diffractometer radius, a 0.02° step size and a 5°/min step time, and microstructures were studied by both a JEOL JSM-7100F scanning electron microscope (SEM, JEOL Ltd., Tokyo, Japan) with an Inca energy 350 energy dispersive spectrometer (EDS, JEOL Ltd., Tokyo, Japan) at 15 kV accelerating voltage, a high-and-low position electron detector and a JEM-2100 transmission electron microscope (TEM, JEOL Ltd., Tokyo, Japan) at 200 kV. 

Hardness was tested on a HV-1000 type micro-hardness tester with a 0.245 N loading force and a 15 s loading duration. Wear test was conducted at room temperature without lubricant on a HT-1000 high temperature ball-on scheme in which a *ϕ*6 mm GCr15 ball was kept on the Cr–Ni sample at a 1.5 N load at a 300 RPM speed for 10 min. Electrochemical performance was examined by a conventional three-electrode cell CHI760C workstation with 3.5 wt.% NaCl (0.6 M) electrolyte solution, and polarization curves were obtained. 

## 3. Results

### 3.1. Phase Identification

**Figure 1:**
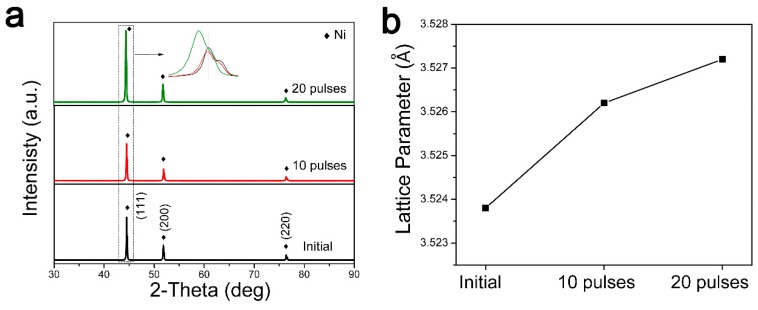
(**a**) XRD patterns and (**b**) lattice parameters of initial pure Ni, 10-pulse and 20-pulse HCPEB-irradiated Cr-Ni samples.

Figure 1 shows XRD patterns and lattice parameters of the initial pure Ni sample, as well as 10- and 20-pulse HCPEB-irradiated Cr-Ni samples. From Figure 1a, the irradiated Cr–Ni samples showed Ni peaks only, which implied that no new phase was generated. Furthermore, from a closer observation of the Ni (111) peaks in Figure 1a, the irradiated peaks had moved slightly to the left compared to those of the initial sample. Moreover, based on a function ($0.5\left(\cot^{2}\theta +\cot\theta \cdot \cos\theta \right)$), the lattice parameters were computed and are graphed in Figure 1b, and it was seen that the lattice parameters of HCPEB-irradiated Cr-Ni samples were higher than those of the initial sample. The lattice parameter of the 20-pulse sample was about 3.5272 Å and, compared to the initial sample, the value increased by 0.0034 Å. According to Bragg’s law (2dsinθ=λ), an increase in the lattice parameter indicates a rise in the plane spacing. Therefore it was proposed that during the non-equilibrium HCPEB irradiation process, some of the Cr atoms diffused into the Ni lattice and replaced the Ni atoms. In addition, since the radius of the Cr atom is larger than that of the Ni atom, the Ni lattice parameter consequently increased [14].

It should also be noted that an obvious increase of the peak width was observed with the 20-pulse irradiated sample, which was in good agreement with other studies [15,16,17,18]. This was because HCPEB irradiation induced rapid melting/remelting on the surface, which resulted in an ultra-fine microstructure, leading to an increase of the peak width. 

### 3.2. Microstructure Analysis

**Figure 2:**
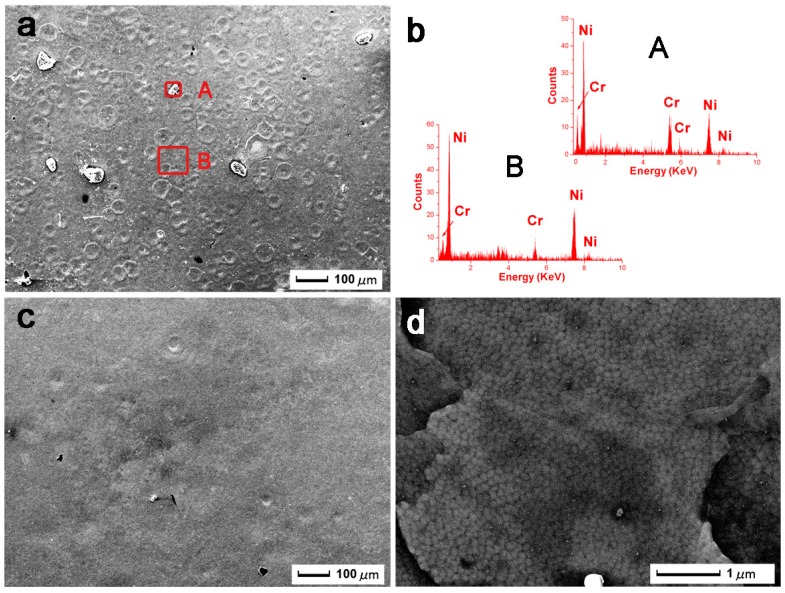
The SEM images of HCPEB Cr-Ni irradiated samples (**a**) 10 pulses, (**b**) energy dispersive spectrum analysis, (**c**,**d**) 20 pulses.

Figure 2 shows SEM images of the 10- and 20-pulse HCPEB-irradiated Cr-Ni samples. From Figure 2a, numerous volcanic-like craters were seen, which was evidence that melting has occurred on the surface. From literature, these typical volcanic-like craters are often observed on HCPEB-irradiated sample surfaces [10,19,20,21]. During HCPEB irradiation, the sample surface firstly started to melt, and the melting liquid rapidly bulged and erupted through the surface. The subsequent rapid cooling kept the surface from fully recovering, which eventually led to the morphology of volcanic-like craters on the surface [22]. Furthermore, some particles were observed, which EDS detected as being Cr particles in Figure 2b area A, and mainly Ni with minor Cr elements in area B. Hence it was inferred that the Cr elements dissolved into the Ni substrate after HCPEB irradiation. When the pulse number increased to 20 as illustrated in Figure 2c, the sample surface became flatter and smoother, and very few craters remained. A better resolution SEM image is given in Figure 2d, and the nanoscale grain size was statistically averaged from 250 nanocrystallines by measuring software, obtaining a value of 124±21 nm. 

These phenomena can be explained by the following mechanisms: During HCPEB irradiation, high energy beams rapidly hit and deposited onto the sample surface, which induced rapid melting. As soon as each irradiation pulse stopped, the heat generated by electron beam quickly dissipated through substrate material, due to the high thermo-conductivity (91 W m^−1^K^−1^) of Ni, which resulted in rapid cooling [23]. This rapid cooling led to a fast solidification of the prior melted surface, which hindered the grain from growing and formed nanocrystalline microstructures. These nanocrystalline grains provided high-density grain boundaries [24,25], which yielded abundant rapid/short diffusion paths for atoms. 

**Figure 3:**
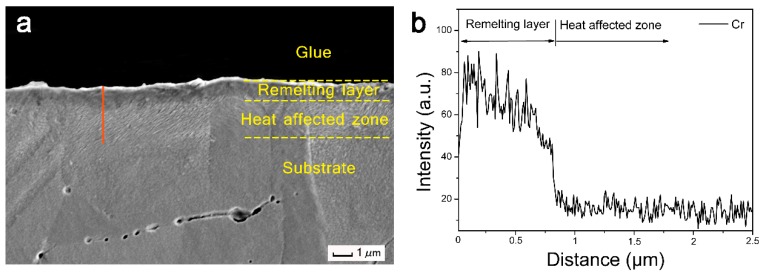
(**a**) A cross-section SEM image and (**b**) EDS line scanning analysis of the 20-pulse HCPEB-irradiated Cr–Ni sample.

Figure 3 is the cross-section SEM image and EDS analysis of the 20-pulse HCPEB-irradiated Cr-Ni sample surface. From Figure 3a, the surface can be categorized into three parts, which from top to bottom are the remelting layer, the heat affected zone, and the substrate. The remelting layer is smooth and thin, with a thickness of less than 1 μm. Underneath is the heat affected zone, in which the microstructure is homogeneous with a thickness of approximately 1.5–2 μm. The bottom zone is the unaffected pure Ni substrate. Figure 3b is the EDS line analysis of the vertical red line shown in Figure 3a, and it revealed that Cr elements were found within the irradiation layer (about 1 μm in thickness), which was approximately the same thickness of the remelting layer. This indicated that the HCPEB irradiation induced the Cr elements to dissolve nicely into the Ni substrate surface, and a Cr-rich alloying layer was formed. 

**Figure 4:**
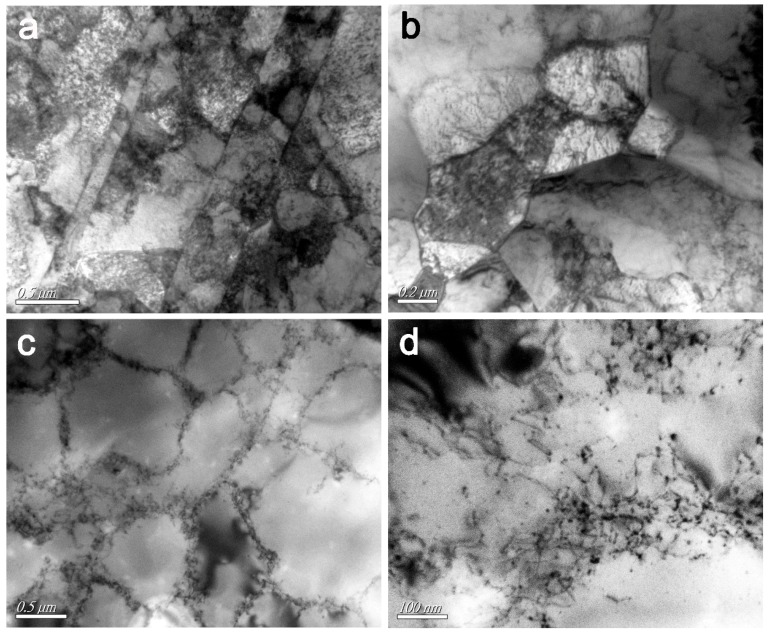
TEM micrographs of the 20-pulse HCPEB-irradiated Cr–Ni alloying layer (**a**) Twins, (**b**) fine grains, (**c**) dislocation cells, (**d**) dislocations.

Figure 4 shows TEM micrographs of the 20-pulse HCPEB-irradiated Cr–Ni alloying layer, where a variety of high-density nanocrystalline defects were found, illustrating that the HCPEB irradiation induced severe plastic deformations. Figure 4a,b shows the twin and nanocrystalline grains respectively. Dislocation lines were found within the grains, and dislocation cells were formed in some areas as shown in Figure 4c. Moreover, it can be seen that the dislocations gathered/aggregated around the borders of dislocation cells/walls as shown in Figure 4d, and some tiny precipitates were observed at the dislocation lines and surroundings. This might be ascribed to the Cr precipitation occurring during rapid heating, whereby grain boundaries and defects have a higher atomic energy, so dissolved Cr atoms are more likely to gather at dislocations and boundaries [26].

**Figure 5:**
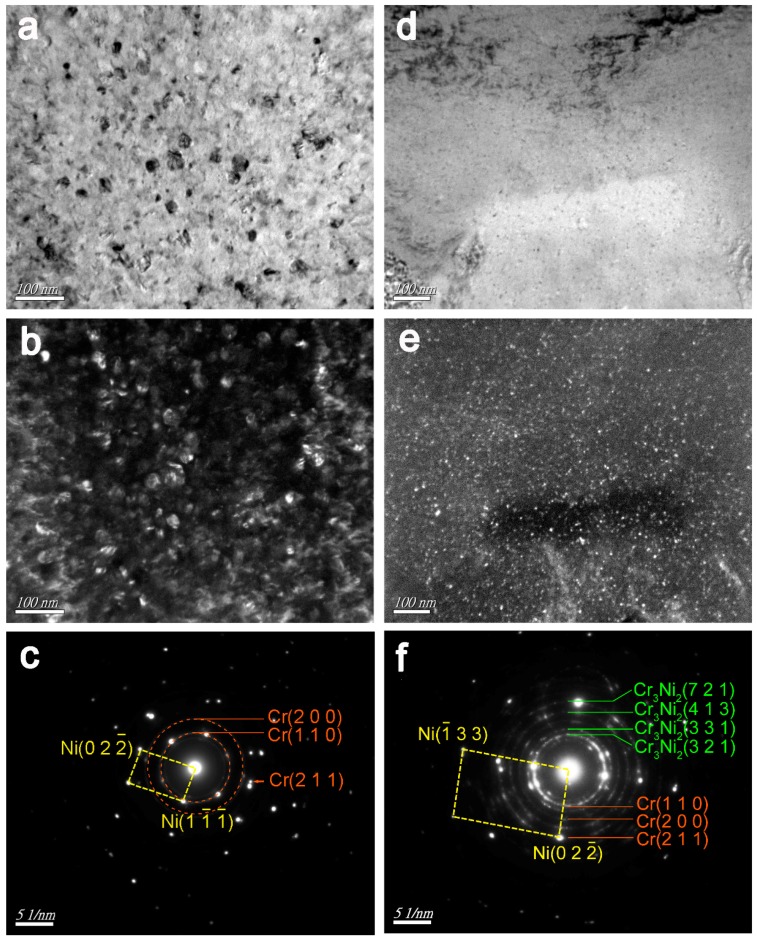
TEM micrographs of the HCPEB-irradiated Cr-Ni alloying layer of 10 pulses (**a**) bright field, (**b**) dark field, and (**c**) the corresponding SAED; 20 pulses (**d**) bright field, (**e**) dark field, and (**f**) the corresponding selected area electron diffraction (SAED).

Figure 5a–c shows the TEM bright field, dark field, and the corresponding SAED images of the 10-pulse HCPEB-irradiated Cr-Ni alloying layer respectively. Plenty of nanoscale particles were observed and indexed as Cr particles. These Cr particles were evenly distributed and approximately 23.6 nm in size. Likewise, Figure 5d–f shows the TEM bright field, dark field, and the corresponding SAED images of the 20-pulse HCPEB-irradiated Cr-Ni alloying layer respectively, and these observed nanoscale particles were also found to be Cr particles approximately 3.5 nm in size. It is thought that the effect of solid solution strengthening amplifies as particle size decreases. Therefore, the solid solubility of Cr in the Ni matrix rose as the HCPEB pulse number increased, which is in good agreement with the work of Guan [27]. Moreover, an intermetallic compound, Cr_3_Ni_2_, was also found in the 20-pulse alloying layer, which is also in good agreement with previous investigations [28,29]. 

In short, during HCPEB irradiation, Cr atoms were dissolved into the Ni matrix, which formed Ni (Cr) solid solution and intermetallic compound Cr_3_Ni_2_. 

### 3.3. Surface Hardness

**Figure 6:**
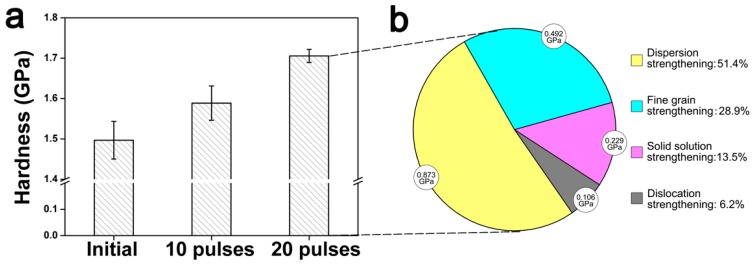
(**a**) Micro-hardness measurements of the initial pure Ni and HCPEB-irradiated Cr­–Ni sample surfaces; (**b**) proportion of strengthening mechanisms of the 20-pulse HCPEB-irradiated Cr–Ni alloying layer.

Figure 6a illustrates a surface hardness histogram of the initial pure Ni and HCPEB-irradiated Cr-Ni samples, and it was observed that the hardness value rose as the pulse number increased due to solid solution strengthening. The highest value of 1.70 GPa was found with the 20-pulse sample (increased by 14% as compared to that of initial sample). The strengthening mechanisms can be attributed to two aspects. The first is the ultra-fine nanoscale Ni and Cr grains, and the second is the over-saturated Cr solid solution. The discussion below focuses on quantifying the strengthening mechanisms of the HCPEB-irradiated Cr-Ni sample surface. 

As shown in Figure 4, numerous defects such as dislocations, dislocation walls/cells and twins, were generated after HCPEB irradiation; therefore the number of crystallographic boundaries, including twin boundaries and refined grain boundaries, multiplied. These defects stimulated the Cr atoms diffusion, which increased the solid-solubility of the Ni(Cr) solid solution. During HCPEB, since the radius of Cr atom is larger than that of Ni, when the Cr atoms dissolved into the Ni lattice interstitial sites distortion of the Ni lattice occurred. This lattice distortion hindered dislocation movements, hence the hardness was improved. This occurrence is known as the solid solution strengthening effect. At the moment, there is no direct method to quantify the solid solution strengthening effect; therefore, in this study a backward approach was adopted. All possible strengthening mechanisms contributing to the hardness value were calculated, and the rest was attributed to solid solution strengthening. 

According to the works of Maruyama and Edalati [30,31], the dislocation strengthening could be represented as yield stress $\sigma_{\rho }$, of which the formula was given as $\sigma_{\rho }=0.5MGb/\rho_{f}{}^{1/2}$, where *G* is the shear modulus (79 GPa), *b* is the magnitude of the Burgers vector (0.2492 nm) and *ρ*_f_ is the dislocation density (in the current study the value was computed as 0.14 × 10^14^ m^−2^) [30,31]. Finally, the value of *σ_ρ_* was obtained as 0.106 GPa (6.2%), which is regarded as the part of the hardness value contributed by dislocation strengthening (also known as strain hardening).

Furthermore, ultra-fine nanoscale grains were formed after HCPEB irradiation, which caused fine grain strengthening. This strengthening can be explained by the nanocrystalline structure [30,32,33,34,35] and represented as yield stress *σ*_fg_ ($\sigma_{\mathrm{fg}}=\sigma_{0}+Kd^{-1/2}$, where *σ*_0_ is the lattice frictional stress (39 MPa), d is the nanocrystalline width (measured as 124±21 nm in current study) and K is the Hall-Petch constant (0.16 MPam^1/2^)). Overall, the contribution of fine grain strengthening is 0.492 GPa (28.9%).

Moreover, after HCPEB, these nanoscale Cr particles were dispersed into the Ni matrix, and the strength of the material was significantly enhanced. This is called dispersion strengthening (also well-known as precipitation hardening). The Cr particles’ dispersion strengthening could be quantified based on the Orowan stress *σ*_or_ [30], $\sigma_{\mathrm{or}}=0.8MGb/\lambda_{\mathrm{or}}$, where M=3 is the Taylor factor, and *λ*_or_ is the mean inter-particle spacing (analyzed as 54.1nm in the current study). The calculated *σ*_or_ value was 0.873 GPa (51.4%).

Thus, the quantity of solid solution strengthening could be calculated from taking out the three strengthening contributors (dislocation, dispersion and fine grain) from the hardness value (1.7 GPa), as graphed in Figure 6b, which illustrates the proportion of each individual strengthening mechanism. It was observed that dispersion strengthening was the dominant contributor (51.4%), while solid solution strengthening played an important role (13.5%) in surface hardening as well.

### 3.4. Tribological Properties

**Figure 7:**
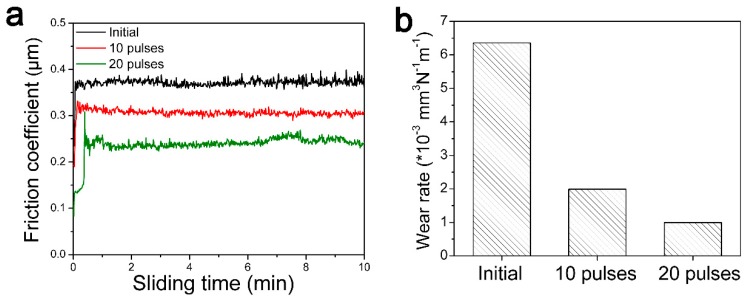
(**a**) The friction coefficient and (**b**) wear rate of the initial pure Ni and the HCPEB-irradiated of Cr–Ni sample surface.

Figure 7 represents the friction coefficient (COF) and wear rate of the initial pure Ni and HCPEB-irradiated Cr-Ni sample surface. From Figure 7a, the COF of the Ni sample was found to be 0.37, and this COF value was reduced after HCPEB irradiation. The COF of the 10-pulse irradiated sample is 0.31, and the value of the 20-pulse sample is 0.25. A trend was found that the COF value decreased as the HCPEB pulse number increased. 

Figure 7b shows the wear rate of the initial pure Ni and the HCPEB-irradiated Cr-Ni sample surface, and likewise, a decreasing COF value is shown as the pulse number increases. The wear rate of the 20-pulse HCPEB-irradiated Cr-Ni sample was 0.99 × 10^−3^ mm^3^N^−1^m^−1^, which was approximately lower than one sixth that of the initial Ni sample. To sum up, the Cr–Ni alloying layer induced by HCPEB irradiation demonstrated excellent tribological properties.

**Figure 8:**
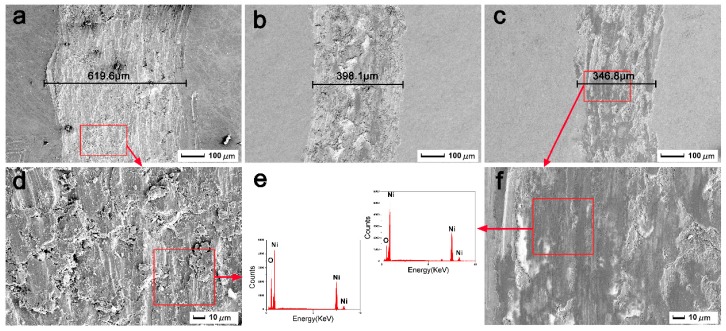
SEM wear profile of the (**a**,**d**) initial pure Ni, and (**b**) 10-pulse, (**c**,**f**) 20-pulse HCPEB-irradiated Cr–Ni sample surfaces; (**e**) EDS line.

SEM wear profiles of the initial pure Ni and the HCPEB-irradiated Cr-Ni sample surfaces are shown in Figure 8, and in which the chemical composition of the worn debris is also given. From the Figure 8a, the width of the wear trace of the initial sample was found to be 616.6 μm. For the irradiated sample, these width values for the 10-pulse and the 20-pulse HCPEB-irradiated sample surface were found to be approximately 398.1 μm and 346.3 μm, respectively, as shown in Figure 8b,c. With a better resolution image of the Ni sample shown in Figure 8d, severe wears and tears were observed, and lots of peeling fragments and abrasive debris/particles were seen. EDS analysis revealed that Ni and O elements resided in the debris as shown in Figure 8e, and crackles were found as well. These findings indicated that the wear mechanism was principally the abrasive wear. 

On the other hand, from the higher resolution SEM image of the 20-pulse HCPEB-irradiated Cr-Ni sample surface shown in Figure 8f, the surface was found to be rather smooth and flat, and the few shallow scratches and debris were seen to be without cracking, which implied that the wear mechanism here was adhesive wear. EDS analyses demonstrated that oxidation occurred on the surface, and the best tribological properties were found with this 20-pulse sample surface. 

For the initial pure Ni sample, the Ni matrix was first deformed and extruded via massive deformation during the initial sliding contact, and then a large amount of debris was fractured or pulled out from the surface, which became more vulnerable to oxidization due to the generation of frictional heat by the friction of coupled parts during sliding. Hereafter, owing to the weaker bonding force between the oxide debris and the Ni matrix, the debris rapidly fragmented or fell off, resulting in a non-continuous oxide layer. Consequently, the initial sample was subjected to dramatically wear, so its COF is higher than those of the irradiated samples. 

For the irradiated sample, it was accepted that the increase in hardness was mainly due to the fact that the Cr elements were uniformly diffused into the Ni matrix. Thus, during the wear test, the irradiated Cr–Ni sample surface kept debris from directly falling off, and consequently the damages caused by wear particles was restrained, leading to a rather smooth and flat wear profile. 

Meanwhile, oxidation occurred on the continuous tribo-surface, forming a thick mechanical oxide layer, which can serve as a protective film or lubricant layer [36]. Therefore, the 20-pulse HCPEB-irradiated Cr–Ni sample surface exhibits an outstanding tribological property in the friction and wear test.

### 3.5. Electrochemical Property

**Figure 9:**
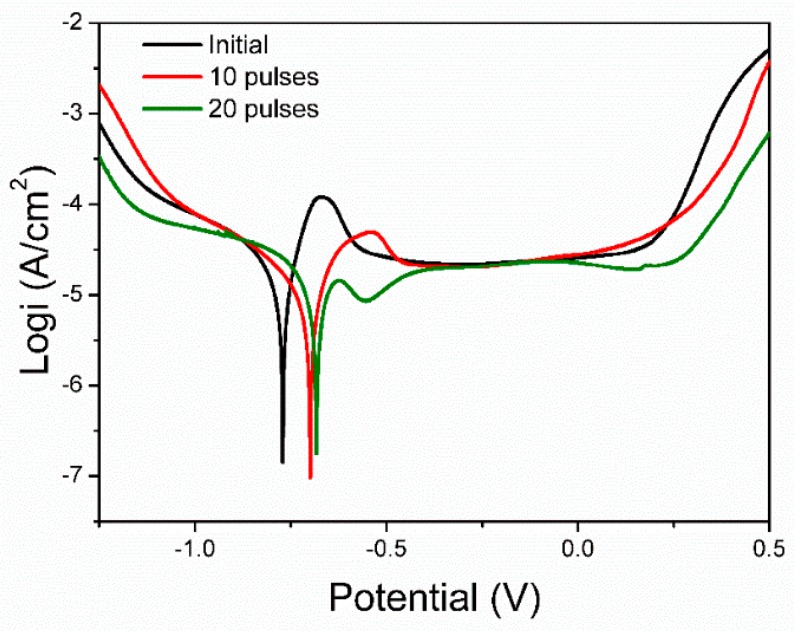
The polarization curve of the initial and HCPEB-alloyed Cr–Ni samples in 3.5 wt.% NaCl solution.

Figure 9 is the polarization curves of the initial pure Ni and the HCPEB-irradiated Cr-Ni samples in 3.5 wt.% NaCl solution. Via Tafel extrapolation, corrosion current density (*i*_corr_) and corrosion potential (*E*_corr_) were computed and listed in Table 2. The corrosion current density, *i*_corr_, indicates the corrosion rate of the material, and the corrosion potential, *E*_corr_, indicates the thermodynamic potential and trends. From Table 2, the *i*_corr_ of the initial sample was 12.59 μA cm^−2^, and after HCPEB irradiation this value fell significantly. The *i*_corr_ of the 20 pulse sample was 3.98 μA cm^−2^, which means that the corrosion rate of the irradiated Cr–Ni sample was much lower than that of the initial one. Moreover, the corrosion potential, *E*_corr_, showed a rising trend when the number of HCPEB irradiation pulses increased. These results clarify that the corrosion property was significantly improved after HCPEB, and that the best anti-corrosion performance was found with the 20-pulse sample. 

For the initial pure Ni sample, corrosion invaded via the dissolution of Ni on the surface, which formed the oxide film (NiO). In Cl^−^ ion solution, when the corrosion potential exceeded the breakdown potential, the dissolution of oxide film (NiO) occurred, which incurred pitting corrosion on the resultant passive film. Subsequently, the passive film was re-formed and then re-dissolved, which yielded the continuous dissolving of the sample surface, and finally the corrosion took place. In this case, the main cause of corrosion was the Ni oxidation. 

For the 20-pulse HCPEB-irradiated Cr-Ni sample, the Ni and Cr oxidation together formed a passive film, which functioned as a protection. During electrochemical testing, abundant nanocrystalline defect structures induced by HCPEB irradiation provided diffusing paths for electrolytic solution and the dissolved O^2−^ ions in the alloyed layer. At the early stages of corrosion, the dissolving O^2−^ stimulated the Ni and Cr elements to rapidly form a passive film (Cr_2_O_3_/CrOOH and NiO) on the sample surface [37,38]. Simultaneously, while the electrolytic solution dissolved into the irradiated surface, corrosive anions (Cl^−^) were brought in, which had a destructive effect. However, because of the formation of the passive film, the effect of the corrosive anions (Cl^−^) on the oxidation film could be neglected [39,40]. Therefore, Cl^−^’s threat was limited, so a thicker, denser and more stable passive film was formed on the surface. As a consequence, the corrosion resistance of the irradiated Cr-Ni sample surface was much better than that of the initial Ni.

## 4. Conclusions

The HCPEB irradiation was successfully conducted on a Cr powder-coated Ni substrate, and an approximately 1 μm thick Cr–Ni nanocrystalline alloying layer was formed on the irradiated surface. The HCPEB-irradiated Cr–Ni sample surface exhibits higher micro-hardness values and better wear properties, and the performance of these properties was improved as the HCPEB pulse number increased. For the improvement of hardness, all the strengthening mechanisms, including dislocation, fine grain, dispersion, and solid solution strengthening were mathematically analyzed and the effect of solid solution strengthening was found to be significant. The corrosion property of the Cr–Ni irradiated sample was also improved as compared to that of the initial Ni, and the best value was found with the 20-pulse irradiated sample. Both the modified nanocrystalline microstructure and the Cr alloying effect contributed to the formation of a denser and more stable passive film, which was the key factor to the enhancement of the anti-corrosion performance. 

## Figures and Tables

**Table 1:** Parameters of the high-current pulsed electron beam (HCPEB) process.

Accelerated Voltage	Current Pulse Duration	Energy Density	Beam Diameter	Pulse Interval	Vacuum
27 keV	1.5 μs	4 J cm^−2^	60 mm	10 s	5 × 10^−3^ Pa

**Table 2:** The corrosion data of the unalloyed and alloyed samples.

Samples	*i*_corr_/(μA cm^−2^)	*E*_corr_/V
Initial	12.59	−0.768
10 pulses	6.31	−0.698
20 pulses	3.98	−0.679
